# Enhancer trapping in zebrafish using the *Sleeping Beauty *transposon

**DOI:** 10.1186/1471-2164-5-62

**Published:** 2004-09-03

**Authors:** Darius Balciunas, Ann E Davidson, Sridhar Sivasubbu, Spencer B Hermanson, Zachary Welle, Stephen C Ekker

**Affiliations:** 1 Arnold and Mabel Beckman Center for Transposon Research, Department of Genetics, Cell Biology and Development, University of Minnesota, 6-160 Jackson Hall, 321 Church St SE, Minneapolis, MN-55455, USA; 2 Molecular, Cellular, Developmental Biology and Genetics Graduate Program, University of Minnesota, USA

## Abstract

**Background:**

Among functional elements of a metazoan gene, enhancers are particularly difficult to find and annotate. Pioneering experiments in *Drosophila *have demonstrated the value of enhancer "trapping" using an invertebrate to address this functional genomics problem.

**Results:**

We modulated a *Sleeping Beauty *transposon-based transgenesis cassette to establish an enhancer trapping technique for use in a vertebrate model system, zebrafish *Danio rerio*. We established 9 lines of zebrafish with distinct tissue- or organ-specific GFP expression patterns from 90 founders that produced GFP-expressing progeny. We have molecularly characterized these lines and show that in each line, a specific GFP expression pattern is due to a single transposition event. Many of the insertions are into introns of zebrafish genes predicted in the current genome assembly. We have identified both previously characterized as well as novel expression patterns from this screen. For example, the ET7 line harbors a transposon insertion near the *mkp3 *locus and expresses GFP in the midbrain-hindbrain boundary, forebrain and the ventricle, matching a subset of the known FGF8-dependent *mkp3 *expression domain. The ET2 line, in contrast, expresses GFP specifically in caudal primary motoneurons due to an insertion into the poly(ADP-ribose) glycohydrolase (PARG) locus. This surprising expression pattern was confirmed using *in situ *hybridization techniques for the endogenous PARG mRNA, indicating the enhancer trap has replicated this unexpected and highly localized PARG expression with good fidelity. Finally, we show that it is possible to excise a *Sleeping Beauty *transposon from a genomic location in the zebrafish germline.

**Conclusions:**

This genomics tool offers the opportunity for large-scale biological approaches combining both expression and genomic-level sequence analysis using as a template an entire vertebrate genome.

## Background

Human, mouse and rat genomes likely have less than 40 000 genes each [1-4]. This is only two to three times as many genes as in *Caenorhabditis elegans *and *Drosophila melanogaster*, and only six times as many as *Saccharomyces cerevisiae *[5-7]. The increased complexity of vertebrates therefore can not be simply accounted for by a larger gene number. A part of the increased complexity is thought to be accomplished by alternative splicing, RNA editing and the use of protein modifications to generate a variety of protein products from a single gene, but everything starts with increased complexity at the level of transcriptional regulation. While promoters are relatively simple and short in yeast, their complexity increases in multicellular organisms, making regulatory sequences ever harder to identify. In humans, enhancer elements can be located over a megabase away from the transcriptional start site [8]. Furthermore, current gene prediction programs used to annotate genomes often fail to correctly identify the 5' start site of a transcription unit, making *in silico *analysis of the regulatory sequences even more complex. To further complicate the matter, enhancer sequences diverge in evolution, co-evolving with their respective transcription factors, and often do not work across large evolutionary distances – worm to fly, for example [9]. This makes information from non-vertebrate model systems sometimes inapplicable to vertebrate sequences.

Enhancer detection ("trapping") using insertion site context vectors was popularized as a genomics tool in *Drosophila*. The first fly enhancer trap vectors were based on the P element transposon and often used the transposase's own promoter fused to the beta-galactosidase reporter gene for enhancer detection [10-12]. Several of the enhancer trap lines were shown to express the *Lac*Z reporter in cells corresponding to the expression patterns of nearby genes, validating the approach [12,13]. In other work, promoters such as *engrailed, fushi tarazu *and *Hsp70 *were successfully developed for enhancer trapping in the fruitfly [14-16]. Further modifications to the system included the implementation of a bipartite system with a Gal4 transactivator [17], green fluorescent protein (GFP) [18], and even a GFP-*Lac*Z fusion protein [19] as reporters. In addition to the P element, other transposons such as *hobo *and *piggyBac *with insertion site preferences distinct from those of the P element have been used in *Drosophila *[20,21]. The availability of a variety of transposons, promoters and reporters for enhancer trapping in the fruitfly enabled researchers to obtain enhancer trap insertions into a considerable fraction of *Drosophila *genes (reviewed by [22]) and allows an investigator to choose vectors most suitable for the problem at hand.

The ability to excise from a genomic location has been instrumental to the utility of P element based vectors. For mutation-causing insertions, reversion of the mutant phenotype by P element excision proves that a given insertion causes the mutation. Since the mutagenicity of *Drosophila *enhancer trap transposons is not significantly higher than the average 15% rate obtained with regular P elements, most insertions do not result in a mutation [22]. In these instances, the P element's ability to induce genomic deletions by "imprecise excision" can be used to obtain a mutation in the neighboring gene(s) [23].

The success of enhancer trapping in *Drosophila *prompted application of this approach in the mouse [24-27]. As was the case in *Drosophila*, the *lac*Z reporter was shown to be expressed in part of the target gene's expression domain [28]. Despite the considerable success of these early experiments, enhancer detection as an experimental approach in mouse was not explored further, giving way to different versions of gene traps (for a review, see [29]).

We believe the success of enhancer trapping in *Drosophila *can be largely attributed to the advantages of this experimental system over mouse. In *Drosophila*, large numbers of transgenic organisms can be readily generated and screened for gene expression patterns. It is far less practical in the mouse. This is partly due to the availability of efficient and precise transgene delivery tools in the fruitfly: the native P element, *hobo *and *piggyBac *transposons. In contrast, early mouse experiments were carried out by non-facilitated DNA transgenesis. This approach is less efficient and prone to induce deletions and other genome rearrangements in the recipient locus, as noted in the first published mouse enhancer trap locus [25,30]. The compact nature of the *Drosophila *genome also contributed to the success of enhancer trapping, making the path from an enhancer trap insertion to the identification of the affected gene straightforward, especially once the *Drosophila *genome was sequenced.

The zebrafish *Danio rerio *is a vertebrate model system that provides many of the advantages found in invertebrates. A few hundred transparent, externally developing embryos can be obtained from a single pair of fish per week. The zebrafish genome is about two-fold smaller than the mouse genome, and its sequencing and annotation are nearing completion. Finally, transposon tools for efficient and precise transgene delivery into the zebrafish genome are available. We focused our research on the *Sleeping Beauty *(SB) transposon system [31,32]. While not as efficient as the highest titer retrovirus used in zebrafish [33,34], the *Sleeping Beauty *transposon system offers advantages in expression as well as ease of construction and testing of diverse vectors that can be done using basic molecular biology tools. Furthermore, the SB system offers the possibility of transposase-induced excision out of the genome to induce local deletions or to revert possible mutant phenotypes.

In this report, we investigated the potential of the SB transposon system for enhancer detection in zebrafish. Our results indicate that zebrafish enhancer trap lines with diverse GFP expression patterns can be readily generated using the SB system. Most of the obtained lines harbor a single transposon insertion event, facilitating the rapid identification of transposon insertion sites responsible for specific GFP expression patterns. We show that two enhancer trap lines exhibit GFP expression patterns matching the expression patterns of the target genes, and that both expected and novel gene expression patterns can be identified using this genomics tool. We conclude that enhancer trapping using the *Sleeping Beauty *transposon system is a viable experimental approach using as template a vertebrate genome.

## Results

### The *Sleeping Beauty *transposon can detect enhancers in *cis*

**Figure 1:**
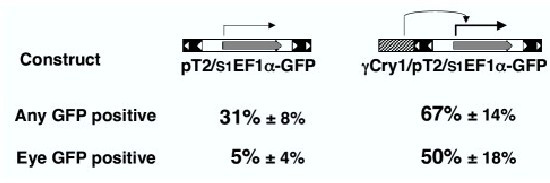
**Artificial enhancer trapping with a *Sleeping Beauty *transposon. **Comparison of GFP expression in embryos injected with pT2/S1EF1α or γCry1/pT2/S1EF1α. Plasmids are diagramed as cartoons on the top of the picture. The SB transposon's inverted repeats are shown as boxes with open triangles, and the GFP open reading frame is depicted as a grey arrow. The gamma-Crystallin promoter/enhancer is shown as a hatched box. DNA-injected embryos which survived to 3 dpf were counted and scored for GFP fluorescence anywhere in the embryo (any GFP) and for fluorescence in the eye (eye GFP), even if there was additional fluorescence elsewhere. The average percentage of embryos positive for particular GFP fluorescence in three independent experiments is shown ± standard deviation.

We have previously established multiple zebrafish lines using SB transposons with ubiquitous and tissue-specific promoters driving reporter expression [32]. Surprisingly, we did not observe any dependency of the expression pattern on the genomic context of the transposon insertion. Multiple studies describing insertion-site dependent transgene expression in vertebrates have suggested that many of those events are due to the transgene falling under control of nearby enhancers [35-40]. For enhancer detection approaches it is imperative that the reporter gene be sensitive to neighboring transcriptional regulatory elements. At least three explanations can be put forward to explain the absence of expression patterns in our previous work in zebrafish. First, the *Sleeping Beauty *transposons are flanked by relatively large inverted repeats. These repeats might function as silencer elements and not allow for transcriptional regulation across them. Second, the promoter we used (*Xenopus laevis *EF1α, [41]) may not be subject to transcriptional regulation by tissue-specific enhancers. Third, the expression level from the selected promoter may be too high to be effectively modulated, as enhancer traps usually contain attenuated promoters. To test these hypotheses, we decided to produce an artificial enhancer trapping event by cloning a tissue-specific promoter / enhancer just outside the inverted repeats and test for an increase in tissue-specific expression in injected embryos (Figure 1). We started with the transposon used in our previous work, pT2/S1EF1α-GM2, which contains a shortened version of the *Xenopus laevis *EF1α promoter driving the GM2 version of GFP in a pT2 transposon vector [32]. We took advantage of the observation that relatively few pT2/S1EF1α-GM2 injected embryos express GFP in the eye. We added a lens-specific *Xenopus laevis *γ1-Crystallin promoter [42] to the pT2/S1EF1α-GM2 vector, as we had previously shown that this promoter specifically expresses in the lens of injected (F0) and transgenic (F1) zebrafish [32]. Embryos injected with pT2/S1EF1α-GM2 or γ Cry1/pT2/S1EF1α-GM2 were scored for any GFP fluorescence and for eye-specific GFP fluorescence at 3 dpf (Figure 1). The addition of γ Cry1 to pT2/S1EF1α-GM2 caused a modest (two-fold) increase in injected embryos showing any GFP fluorescence. In contrast, the increase in eye-specific GFP expression was ten-fold (Figure 1). We concluded that at least in this assay, the *Sleeping Beauty *inverted repeat sequences do not block transcriptional regulation and that the EF1α promoter can be subject to transcriptional regulation from external, tissue-specific regulatory sequence elements.

### Promoter truncations and pilot screens

**Figure 2:**
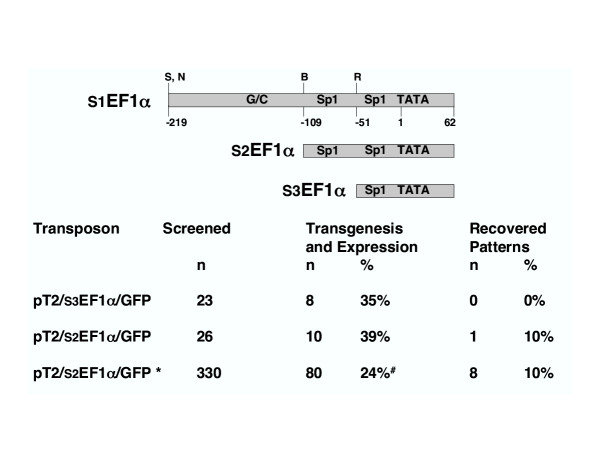
**EF1α promoter truncations and endogenous enhancer trap screening. **A diagram of the S1EF1α promoter [32, 41]. Restriction enzyme sites are shown on top as single letters. S is *Sph*I, N is *Nhe*I, B is *Bst*1107I and R is *Eco*RI. G/C, G and C rich box. Sp1, Sp1-like site. TATA, TATA box. Numbering below is relative to the first T of the TATA box. The table below the diagram shows the results of the pilot and scale-up (*) screens. Transgenesis and expression rates are shown, non-expressing transposon insertions were not scored. Transgenesis and expression rate from scale-up screen (^#^) is an underestimate since many founders were screened by incross and crosses from doubly transgenic founders were scored as a single transmission event (see text).

**Figure 3:**
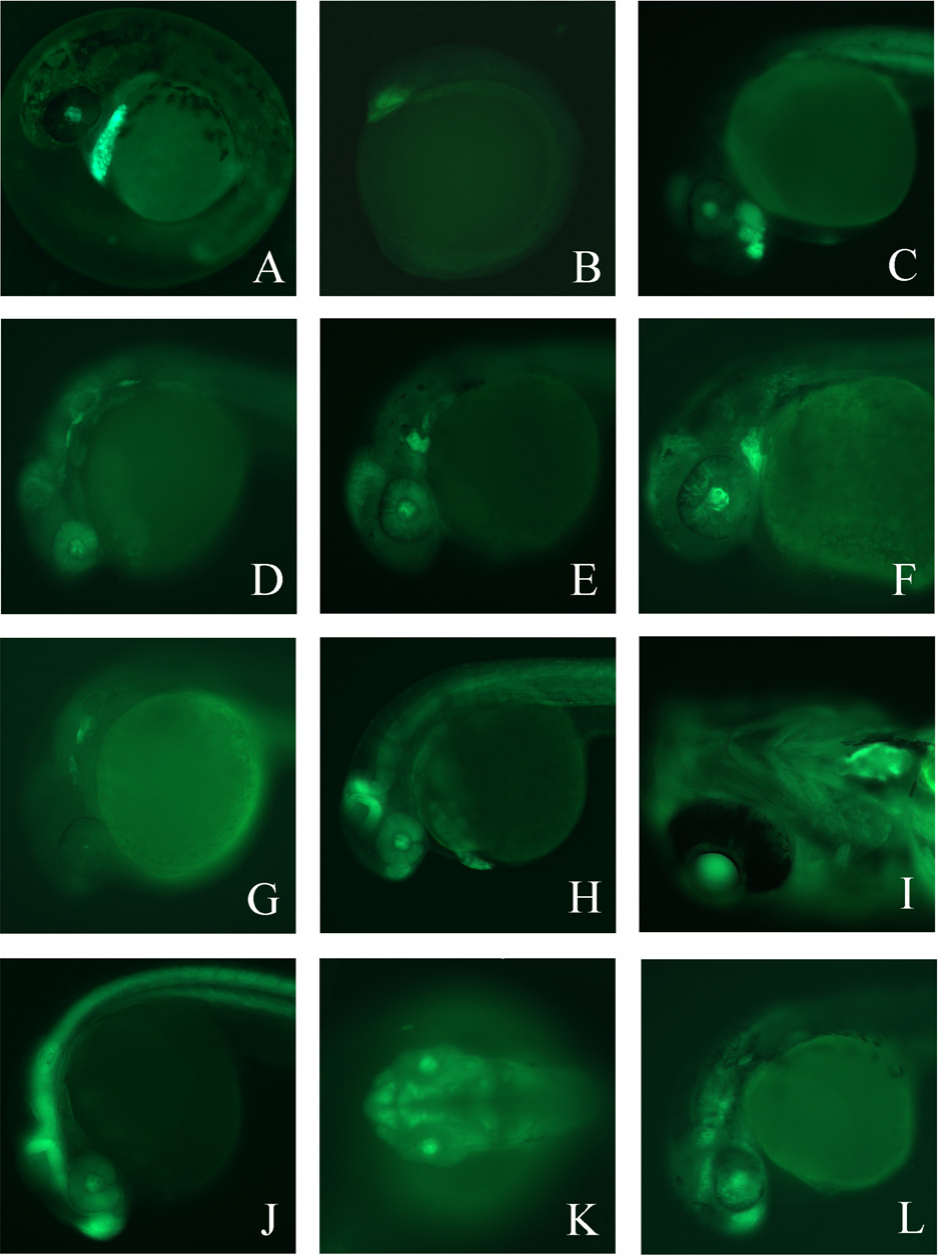
**Enhancer trap lines exhibit a variety of unique GFP expression patterns. **(A). Lateral view of GFP expression in Enhancer Trap line 1 (ET1) at 38 hours post fertilization (hpf). (B) ET3 at 5–6 somite stage. (C) ET3 at 36 hpf. (D) ET4 at 26 hpf. (E) ET5 at 30 hpf. (F) ET5 at 48 hpf. (G) ET6 at 26 hpf. (H) ET7 at 32 hpf. (I) Ventral view of ET7 at 5 dpf. (J) Lateral view of ET8 at 26 hpf. (K) Dorsal view of ET9 at 28 hpf. (L) Lateral view of ET9 at 30 hpf. In all panels, anterior is to the left. See text for details.

We next tested the hypothesis that the absence of expression patterns in our previous work was due to the fact that S1EF1α is expressed too strongly in transgenic animals. Since most successful enhancer traps in *Drosophila *and mice were based on truncated or weak promoters, we decided to attenuate the S1EF1α promoter by removing sequences upstream of *Bst*1107I (S2EF1α) and *Eco*RI (S3EF1α) restriction enzyme sites (Figure 2). We then co-injected the corresponding transposon constructs with SB10 transposase mRNA to assess germline transmission, expression and enhancer trapping rates. In pilot experiments, progeny from over 20 fish were screened with each construct. While overall germline transmission and expression rates were comparable (Figure 2), there was a difference in the expression patterns of the two constructs in transgenic embryos. Most of the transgenic animals generated with pT2/S3EF1α-GM2 exhibited weak GFP expression, and we could not detect any expression patterns (data not shown). In contrast, when pT2/S2EF1α-GM2 was used, most of the GFP-positive fish exhibited fairly strong, ubiquitous expression. Closer analysis indicated that many of these "ubiquitous" expression patterns were rather unique, with GFP expression often noted to be particularly strong in some tissues, consistent with a tissue-specific expression pattern superimposed on a ubiquitous expression pattern (data not shown). In most cases, GFP expression segregated as a single integration event, indicating that a tissue-specific expression pattern was not likely being masked by a ubiquitous expression pattern from a different insertion event. A similar phenomenon has been observed with *Drosophila *enhancer traps [11]. We speculate that in those instances, the GFP expression cassette may have fallen under the control of multiple enhancers – some tissue-specific, some ubiquitous. Alternatively, the ubiquitous expression may stem from the ubiquitous activity of the EF1α promoter used in the screen, with tissue-specific enhancers only elevating the expression levels in certain tissues, but not restricting it. We did not consider such expression patterns valuable and did not establish any fish lines with such GFP expression. Importantly, one of the founders in the pT2/S2EF1α-GM2 pilot screen yielded three kinds of GFP expression in its progeny. Some were ubiquitously GFP positive, some showed a hatching gland-specific expression profile, and some exhibited both. When three F1 fish with both ubiquitous and hatching gland specific expression were raised and outcrossed, the two expression patterns exhibited independent segregation: 24% of the embryos were GFP negative, 26% expressed GFP ubiquitously, 25% had hatching gland-specific GFP expression, and 25% had both hatching gland-specific and ubiquitous expression (n = 245). Independent segregation indicates that the two transposon insertions causing the two expression patterns are unlinked. Two independent integration events were confirmed by Southern hybridization and inverse PCR (data not shown). The hatching gland-specific GFP expressing embryos were used to establish our first enhancer trap line, ET1 (Figure 3A). We concluded from our pilot screens that pT2/S2EF1α-GM2 demonstrated the desired properties for potential use in enhancer trapping studies.

### Germline excision of a *Sleeping Beauty *transposon insertion

We have previously demonstrated the excision of a *Sleeping Beauty *transposon from the genome in somatic tissues of transposase-injected zebrafish embryos [43]. We tested if such an excision event could be inherited by examining transposon excision in the germline. Embryos homozygous for the ET1 insertion were injected with SB10 transposase mRNA, and while some were used for a somatic excision assay the rest were raised to test for germline transmission of an excision event. A PCR reaction on genomic DNA from transposase-injected embryos with primers flanking ET1 insertion point produced two bands. A large band corresponded in size to the transposon insertion allele, and a small band corresponded to a transposon-less allele (data not shown). Both cannonical *Sleeping Beauty *transposon footprints (ATGTCAT and ATGACAT, [44,45]) were obtained upon cloning and sequencing of the smaller band, indicating a transposase-mediated excision and DNA repair. 26 fish were screened for germline transmission (see Materials and Methods), and one was shown to transmit the expected excision footprint. We conclude that the *Sleeping Beauty *transposon can be excised from a genomic location in the zebrafish germline.

### pT2/S2EF1α-GM2 scale-up screening: 10% of GFP-expressing integrations yield tissue-specific patterns

One tissue-specific expression pattern was recovered from our pilot screen. We sought to recover more patterns and to test if enhancer detection in zebrafish is amenable to scale-up. To that end, we co-injected 3248 zebrafish embryos with the pT2/S2EF1α-GM2 and SB10 transposase mRNA mix. 2102 embryos survived to day 3 for scoring, of which 848 were mosaic GFP positive and were selected to be raised. 330 survived to adulthood and were screened for germline transmission of GFP expression, primarily by sibling incrossing. This approach provided a lower estimate of the transgenesis and expression rate because it does not distinguish instances were both parents are transgenic. In this screen, at least 80 of the founder fish produced GFP-expressing progeny resulting in a minimum estimate of a 24% transgenesis rate. The actual transgenesis rate is closer to 30% because most of the fish were screened by incross, and if a pair produced GFP-expressing progeny, only one parent was counted as a transmitter. Eight of the GFP-expressing fish displayed distinct GFP expression patterns (Figure 3). Together with the pilot screen, 9 tissue-specific expression lines were obtained from 90 transgenic founder fish (10%) using the pT2/S2EF1α-GM2 transposon.

### Recovered expression patterns label a diverse array of tissues during embryogenesis

GFP expression in ET1 can be first observed in the polster region at 7–8 somite stage (not shown). The expression is very pronounced between 20 and 40 hours post-fertilization (hpf), when it marks the hatching gland (Figure 3A). Expression disappears as the hatching gland is resorbed. Line ET3 represents a pattern with the earliest onset of expression. Anterior localization of GFP in the diencephalon is detected by 5–6 somite stage in this line (Figure 3B). Extremely bright anterior expression persists in the ventral diencephalon (Figure 3C) and by 6 days post-fertilizations (dpf) is restricted slightly more posterior in the midline. The onset of expression for ET4 is 18 hpf with a bilateral expression pattern in cranial sensory ganglia that remains strong until 2 dpf and is undetectable by 5 dpf. This anterior expression in ET4 seems to label the lateral line ganglia both anterior and posterior to the otic vesicle (Figure 3D), however, no expression is detected in the lateral line in the trunk. In ET5 a single bilateral patch of strong GFP expression in the hyoid arch is observed by 24 hpf (Figure 3E), that by 48 hpf marks a more anterior location in the embryo (Figure 3F). Expression in this line is greatly diminished by 3 dpf and is undetectable by 5 dpf. Strong GFP expression is observed in ET6 by 26 hpf as a bilateral expression pattern consisting of two distinct patches in a subset of cranial sensory ganglia/placodes (Figure 3G). The expression weakens by 2 dpf and is undetectable by 3 dpf. GFP expression in ET7 begins weakly in the midbrain-hindbrain boundary (MHB) at 12–14 somites with the most pronounced expression in the anterior side of the MHB detected by 26 hpf (Figure 3H). Robust expression in the heart is first detected at around 32 hpf and remains ventricle-specific through 5 dpf (Figure 3I), even though expression in the brain is no longer restricted to the MHB. GFP expression in ET8 is already localized by 10–12 somites and remains strong in the telencephalon, and posterior side of the MHB through 26 hpf (Figure 3J). By 3 dpf the localized anterior expression is undetectable over autofluorescence, however, caudal expression appears to be enhanced in the dorsal neural tube. The onset of expression in ET9 occurs around 22 hpf and is difficult to detect by 2 dpf. At 28–30 hpf (Figure 3K,3L), three distinct expression domains are apparent in the telencephalon, diencephalon and hindbrain of ET9.

### The ET2 line expresses GFP specifically in the motoneurons

**Figure 4:**
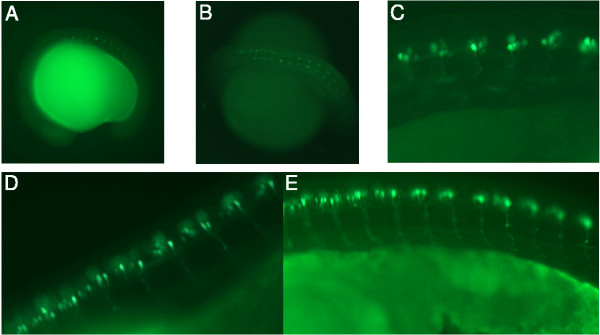
**The ET2 transgenic fish line expresses GFP in caudal primary motoneurons. **GFP expression in ET2 was visualized in motoneurons using a bandpass GFP filter set at various stages of embryonic development. In all panels anterior is to the left. (A) The onset of GFP expression in ET2 line at 16 somite stage. (B) 26 somite stage. (C) 24 hpf. (D, E) 36 hpf. Axonal trajectories are visible at 24 and 36 hpf.

We analyzed the ET2 line in detail because of the highly specific expression of GFP in these fish. GFP expression was first observed at the 16 somite stage, when 2 bilateral cells in the spinal cord of the 10 anterior somites become GFP positive (Figure 4). At later stages, multiple cells per somite become GFP positive, either due to continued expression of GFP mRNA or due to segregation of GFP to daughter cells. GFP expression follows the wave of somitogenesis, with the posterior-most somites lagging in GFP expression. At about 24 hours, ventrally-projecting axons become visible by GFP fluorescence. Later yet a pattern of nodes appears along the axons (Figure 4). Based on the position of neuronal cell body and the axonal trajectory, we conclude that caudal primary motoneurons express GFP in this line [46]. To our knowledge, this is the first gene to be specifically expressed only in this subpopulation of motoneurons. We therefore sought to identify the locus tagged by this transposon insertion.

**Figure 5:**
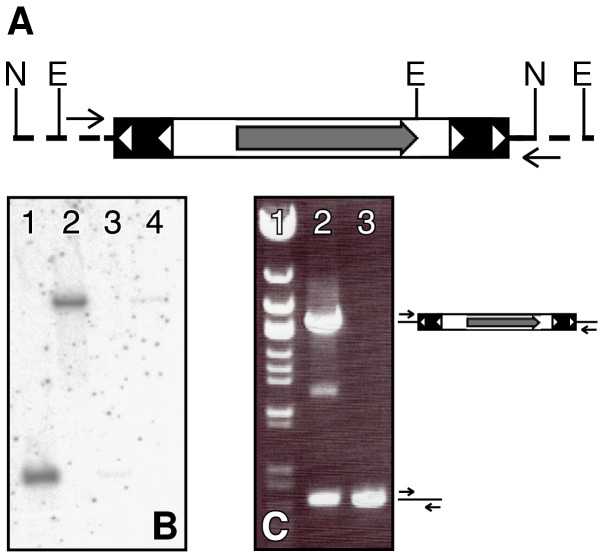
**Identification of the transposition event in the ET2 line. **(A) The pT2/S2EF1α transposon insertion into zebrafish genome is shown; restriction enzyme sites and primers used for molecular analysis are indicated. Transposon IR/DR's are shown as solid boxes with open triangles, and the GFP open reading frame is shown as a grey arrow. Genomic DNA is shown as a dotted line. N is *Nsi*I, E is *Eco*RV. (B) Southern blot on ET2 line outcross embryos. DNA from GFP positive (lanes 1 and 2) and GFP negative (lanes 3 and 4) embryos was digested with *Nsi*I (lanes 1 and 3) or *Eco*RV (lanes 2 and 4) and probed with a GFP-specific probe. (C) Linkage of the transposon insertion event to GFP expression. Primers flanking the transposon insertion event (arrows) were used to conduct PCR on DNA from GFP positive (lane 2) and GFP negative (lane 3) embryos from an ET2 outcross different from the one used in (B). Lane 1, λ *Eco*47III Marker (Fermentas Inc).

Southern analysis indicated that there is a single transposon insertion in this line, and it is linked to GFP expression (Figure 5). Inverse PCR identified a transposase-mediated insertion into a TA dinucleotide at position 256083 on contig ctg9701 (zebrafish genome assembly Zv3). The insertion occurred into a Genescan-predicted gene. Further analysis indicated that the Genescan-predicted gene actually consists of parts of at least two different genes, myoferlin and poly(ADP-ribose) glycohydrolase (PARG). The insertion located in the PARG part of the predicted transcript, 649 nucleotides from an exon just upstream of the PARG catalytic site. To confirm that the transposon insertion into the PARG gene induced GFP expression in primary motoneurons, we prepared genomic DNA from both GFP positive and GFP negative embryos from an independent outcross, and we conducted a PCR with NeuroIns-F1 and NeuroIns-R1 primers specific to the flanking sequences. In GFP negative embryos, a 0.5 kb band corresponding to wild type locus is noted. In GFP positive embryos, the same band is seen in addition to a larger band of approximately 2.4 kb, corresponding to a locus with transposon insertion (Figure 5). Since the inverse PCR and confirming PCR was performed on DNA from different batches of embryos, we can exclude the possibility of DNA contamination or fish husbandry error and conclude that the enhancer trap transposon insertion into the PARG gene causes GFP expression in caudal primary motoneurons.

### GFP expression in ET2 line matches the expression of the endogenous PARG gene

**Figure 6:**
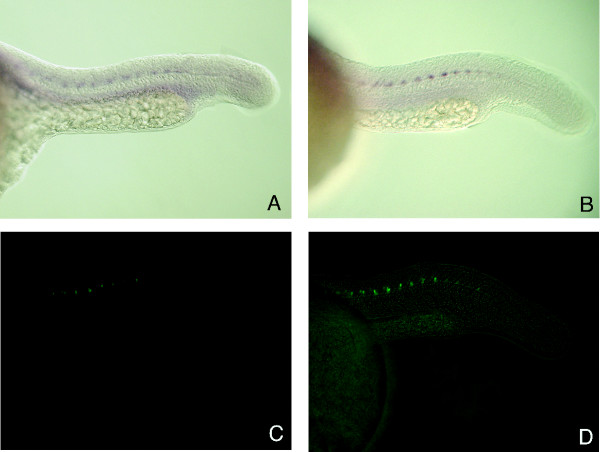
**GFP expression in ET2 line embryos is indistinguishable from endogenous PARG gene expression. **23 hpf embryos collected from a heterozygous outcross were photographed for GFP fluorescence and sibling embryos were fixed for *in situ *hybridization. (A) *In situ *hybridization with PARG antisense probe. (B) *In situ *with GFP antisense probe. (C) Visualization of GFP expression in living embryos using a bandpass GFP filter set. (D) The same embryo as in (C) photographed using a bandpass GFP filter set with a low level of bright field illumination to visualize GFP expression in relative position to the somites.

Poly(ADP-ribosyl)ation is a protein modification that is extensively studied at the biochemical level and is associated with changes in DNA replication, recombination, repair and transcription [47], for a review, see [48]. Recently poly(ADP-ribosyl)ation was demonstrated to have a role in long term memory in the sea slug *Aplysia *[49]. Most organisms have multiple genes for poly (ADP-ribose) polymerases but only a single known gene for poly (ADP-ribose) glycohydrolase [48]. PARG activity is noted to be expressed in many cell lines, among them neuronal [50-53], but the tissue specificity of PARG expression during embryogenesis has not been reported for any organism. To test if the pT2/S2EF1α-GM2 enhancer trap recapitulates the expression pattern of the endogenous PARG gene, we conducted whole mount *in situ *hybridization on ET2 outcross embryos to compare PARG and GFP reporter expression (Figure 6). *In situ *hybridization visualizes axonal cell bodies, the position of which appears indistinguishable with both PARG and GFP probes. We therefore conclude that GFP mRNA expression in this enhancer trap line faithfully recapitulates the expression of the zebrafish PARG gene during embryogenesis.

### Molecular analysis of other enhancer trap lines identifies target genes

We characterized insertion events in other enhancer trap lines. GFP positive F1 or F2 fish were outcrossed, and embryos were sorted into GFP positive and GFP negative pools. Genomic DNA was prepared from each pool, and Southern analysis was conducted to assess transposon copy number and linkage to the GFP expression pattern. In all lines except ET1 (see above), a single GFP expression-linked transposon insertion event was detected by Southern hybridization. We then conducted inverse PCR analysis on the DNA from GFP positive embryos to identify the insertion locus. For verification, DNA from embryos from an independent outcross was prepared and PCR was run with primers flanking the insertion site to link GFP expression and transposon insertion at a particular locus. Verified enhancer trap loci are presented in Table 1. Notably, seven of the insertions have occurred into introns of Genescan-predicted genes. Four of the tagged genes show significant homologies to previously identified genes: PARG (see above), MAPK upstream kinase-binding inhibitory protein (MBIP) [54], a member of cytochrome P450 superfamily and Nidogen [55]. The other three tagged predicted genes do not have significant homologies to previously identified genes. In the two lines which have insertions into intergenic regions, transposons have integrated less than 25 kb from the nearest predicted transcript.

**Table 1:** pT2/S2EF1α-GM2 transposon insertion events in analyzed enhancer trap lines.

Trap line	Sequence	Insertion location	Predicted gene
ET1	ATTGTCCtTAGTG**TA**TGTGTTTGTGTGA	Chr. 4	none
ET2	CAAAAAGACTATA**TA**TAGGAGGCTTCAA	ctg9701	PARG
ET3	AACGCTTACCATG**TA**TGTTAATAAATGT	Chr. 17	MBIP
ET4	TATATCAAAATTA**TA**TATATGAACGTAT	Chr. 6	Cyt. P450
ET5	GTACATAcACATG**TA**CAAATCaACATTA	ctg13471	novel
ET6	ATTTTAAACAAAC**TA**AGTtGAACATTAC	ctg13605	Nidogen
ET7	ATCACAGAGCATC**TA**GCTTGGATGTGCT	ctg12155	novel/*mkp3*
ET8	TATACAACAAACT**TA**TCTAACGTGCAAT	Chr. 2	none
ET9	TATTTAATATATA**TA**TTATATTATATTA	Chr. 19	novel

**Figure 7:**
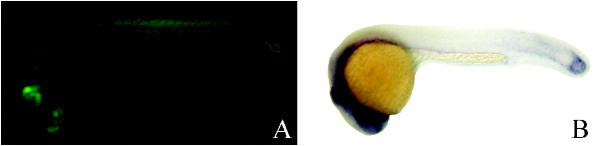
**GFP expression in ET7 line matches *mkp3 *mRNA expression. **(A) GFP fluorescence photograph of an ET7 embryo at 23 hpf. (B) *In situ *hybridization on 23 hpf wild type embryo using *mkp3 *antisense RNA probe.

Left column, line designation. Sequence column, the genomic sequence the transposon has inserted into. The target TA dinucleotide is highlighted in bold. The sequences flanking the left inverted repeat are to the left of the target TA, and sequences flanking the right inverted repeat are to the right of the target TA. Lowercase indicates mismatches between an actual sequence read and the current zebrafish genome sequence (Assembly ZV3). Both left and right transposon/genomic DNA junctions were sequenced for ET1, ET2, ET3, ET5 and ET7. Only the left junction was read for ET8, and only the right junction was read for ET4, ET6 and ET9. Insertion location column, predicted insertion chromosome or contig (Zv3 assembly of the zebrafish genome). Predicted gene column, the gene into which the transposon has inserted as annotated in the zebrafish genome assembly Zv3. Novel indicates no significant homologies. Gene name indicates significant homology to denoted genes. The predicted integration into an intron of the PARG locus for line ET2 was experimentally confirmed (see text). For ET7, a comparison of the observed expression pattern in this line with that of a known nearby gene (*mkp3*) indicates this insertion has most likely trapped an enhancer for this gene (Fig. 7, see text). Actual sequence reads which were used to determine the genomic location of the transposon insertions were longer than shown in this table and are available upon request.

### ET7 line has a transposon insertion near *mkp3 *locus and matches *mkp3 *expression pattern

The transposon insertion in ET7 line has occurred into a predicted novel gene (Table 1). Closer investigation of the target locus revealed the presence of a previously characterized zebrafish *mkp3 *gene within 30 kb of the insertion site. Our attempts to amplify the predicted novel candidate gene from maternal and post-somitogenesis zebrafish cDNA libraries using 2 different primer pairs failed, while *mkp3 *cDNA was readily amplified in parallel PCR reactions (data not shown). This suggests that the novel target gene may be a false prediction by Genescan. The *mkp3 *gene encodes a dual specificity phosphatase which was cloned as a member of *fgf8 *synexpression group and is a negative feedback regulator of FGF8 signaling. *mkp3 *is expressed in the midbrain-hindbrain boundary, forebrain, tailbud, branchial arches, developing ear, pectoral fin buds and other tissues [56,57]. GFP expression in ET7 line closely mimics *mkp3 *mRNA expression pattern in 23 hour embryo (Figure 7). The only significant difference is that GFP expression is stronger in somites and not as bright in the tailbud, even though the tailbud expression becomes brighter at later stages of development (data not shown). We did not observe GFP expression in the pectoral fin buds, even though we reproduced *mkp3 *expression in the fin buds just after after 24 hpf by *in situ *(data not shown, [56,57]. An intriguing possibility is that *mkp3 *expression in pectoral fin buds may be controlled by a different enhancer, one we do not detect in this transgenic line. Additionally, ET7 expresses GFP in the heart after 24 hpf, and the expression clearly localizes to the ventricle at 5 dpf (Figure 3I). Expression of *mkp3 *in the heart after 24 hpf was not reported, and we did not conduct *in situ *hybridization on late pharyngula stage embryos to test for it. However, *fgf8 *is expressed in the ventricle of the zebrafish heart at 36 hpf [58]. Taken together, this suggests that GFP expression in ET7 line mimics a subset of the complete expression pattern of the zebrafish *mkp3 *gene.

## Discussion

In this paper, we describe the first use of enhancer trapping, or enhancer detection, as an experimental approach in zebrafish. We show that *Sleeping Beauty *transposons can trap enhancers by testing an artificial enhancer trapping event *in vivo*. This approach is likely to also be useful in the construction and testing of other trap vectors: gene (5' exon) and polyA (3' exon) and other related constructs. We then constructed two further truncations to the S1EF1α promoter in the transgenesis cassette [32] and found one to be particularly suitable for enhancer trapping. Ten percent (9 of 90) of GFP-expressing transgenic fish generated lines with unique GFP expression patterns. All reagents described in this paper, including the enhancer trap fish strains, are readily available upon request .

Many of the obtained enhancer trap lines express GFP in the nervous system. This was previously observed with both mouse and *Drosophila *enhancer trap vectors and was speculated to stem from the transcriptional complexity of neural tissue [11,28]. Several of our lines also exhibit some level of GFP expression in the eye. At least two explanations can be put forward to explain this observation. First, many genes are expressed in the developing eye. Thus, the eye expression that we see may reflect expression of the tagged genes in the eye. Second, optical properties of the tissues in the eye may permit detection of GFP expression that is lower that what would be required for detection in other tissues.

The ET2 line harbors a transposon insertion into the zebrafish gene for poly(ADP-ribose) glycohydrolase (PARG). We demonstrate that both PARG and GFP in ET2 line are expressed in caudal primary motoneurons of 23 hour old embryos. Thus, GFP expression in the ET2 line mimics that of an endogenous gene (PARG), indicating that transgene expression is under control of an endogenous enhancer. A very intriguing question is what the actual trapped enhancer sequence is, how far away from the genomic enhancer the trap can insert and still detect it, and weather artificial enhancer trap approach (Figure 1) can be used to answer these questions.

The ET7 line has a transposon insertion into a predicted novel gene 30 kb downstream of the zebrafish *mkp3 *locus. GFP expression in that line closely resembles part of the *mkp3 *expression domain, suggesting that the enhancer trap transposon in that line is under control of a subset of *mkp3 *enhancer elements.

Zebrafish enhancer trap lines will be valuable in future developmental genetics studies, be it classical mutagenesis or morpholino "knockdown" screening [59]. GFP expression can be used as a sensitive marker for certain tissue or cell types. For example, the ET1 line expresses GFP in the hatching gland. The expression of the *hgg1 *gene is specific to the polster and hatching gland depends on *nodal *signaling and is absent in *one-eyed-pinhead *mutants [60]. We phenocopied the *one-eyed-pinhead *mutation by morpholino injection in ET1 homozygotes and observed a complete loss of hatching gland-specific GFP expression (data not shown). While the ET1 line expresses GFP in an organ that can be readily observed using regular light microscopy techniques, other lines visualize tissues that are not nearly as easily morphologically accessible. In particular, the ET2 line visualizes the position of primary motoneuron cell bodies and axonal trajectory. Morpholinos against known genes or new members of the zebrafish secretome [61] can be screened for effects on neuronal cell body position or axonal pathfinding in the developing embryos by injection into ET2 line embryos. The ET7 line may provide a fluorescent readout of FGF8 signaling, thus facilitating the identification of genes involved in that signaling pathway.

A further utility offered by the transposon system is the possibility to revert a mutant phenotype or to generate localized deletions by transposon excision [23]. We successfully excised the transposon in the germline of the ET1 line, resulting in the expected transposon footprint. It has been shown that excision of the *Sleeping Beauty *transposon from a plasmid results in local deletions with fairly high frequency which is dependent on the cell type or tissue used [45]. Furthermore, the frequency of imprecise excision of *Sleeping Beauty *transposons significantly increases in cells with a compromised DNA repair pathway [62,63]. It remains to be determined how frequently the excision of a *Sleeping Beauty *transposon from a genomic location in zebrafish germ line is accompanied by a deletion of flanking genomic DNA, and it should be possible to compromise the embryo's DNA repair machinery to induce such deletions at a high frequency.

Our experiments indicate that enhancer detection using *Sleeping Beauty *transposons is an easily scalable and efficient experimental technique in zebrafish. Obtaining fish with different GFP expression patterns is not the rate limiting step in this process. Preliminary molecular analysis of the insertion site is also straightforward using inverse PCR techniques. Identification of candidate genes should benefit from the progress in zebrafish genome sequencing and annotation. The main bottleneck step is the detailed biological analysis of GFP and the corresponding candidate gene expression profile.

In *Drosophila*, the generation of transposase-expressing lines of flies made enhancer detection and P-element mutagenesis in general a mainstream approach. Even without a similar gain in efficiency, transposase expressing fish lines would make enhancer trapping as well as related gene- and poly(A)-trap methodologies even more accessible for high-throughput functional analysis of the vertebrate genome.

## Methods

### Plasmid construction

pT2/S1EF1α-GFP (pDB358) was previously published [32]. To make γ Cry/pT2/S1EF1α-GFP (pDB375), a *Bam*HI-*Hind*III fragment from Cry1-GFP3 [42] containing part of the *X. laevis *γ-Cry1 promoter was cloned into the Ecl136II site of pDB358. To produce pT2/S2EF1α-GFP (pDB371), a part of the EF1α promoter was deleted from pDB358 by ligation of the *Bst*1107I-*Nco*I and *Nhe*I-*Nco*I fragments of pDB358. Similarly, the *Eco*RI-*Nco*I and *Nhe*I-*Nco*I fragments of pDB358 were ligated to produce pT2/S3EF1α-GFP (pDB372).

### Inverse PCR, PCR and primer sequences

For inverse PCR experiments, zebrafish genomic DNA was digested and ligated as described [64]. 1 and 2.5 microliters of the ligation reaction were used for the first PCR reaction with RP1/LP1 or RP1/GFP-R1 primers in total volume of 25 μl. 1 μl of the first PCR reaction was used as a template for the second (nested) PCR reaction with primer pairs RP2/LP2 or RP2/GFP-R2, respectively. Expand Hi Fi PCR system (Roche) was used for all PCR reactions. A MJ Research PTC-100 PCR machine was used for PCR with the following program : 92°C 4 min., 92°C 10 sec., 60°C 30 sec., 68°C 6 min., 30 cycles. Starting at cycle 11, 20 sec. per cycle was added to the extension time. The same PCR reaction with an annealing temperature 55°C was used for amplification with primers flanking transposon insertion sites, and for amplification of partial PARG cDNA from a maternal cDNA library. Primer sequences are: LP1 GTGTCATGCACAAAGTAGATGTCC [32]; LP2 ACTGACTTGCCAAAACTATTGTTTG; nRP1 CTAGGATTAAATGTCAGGAATTGTG; RP2 GTGAGTTTAAATGTATTTGGCTAAG; GFP-R1 TTCGGGCATGGCACTCTTG; GFP-R2 TATGATCTGGGTATCTCGCAA; NeuroC1-F1 CGTAAAGATGCCTTGTTCAGAA; NeuroC1-R1 ATTCCGTGACTCTCCTGAAATA; NeuroIns-F1 GGCTTGCATACATGACTAATG; NeuroIns-R1 GAAGACTGAAGTCCTCAAACT; HG1-1 ACATTGAGCCACTAAGCATTG; HG-2 TGTGTGCACTTAAGGGGCGA. Mkp3-F1 AGTGTTGCATTCTCCAGGATA; Mkp3-R1 TGACACAGAACTTCCCTGAAC; EF1a-F2 TTCCTGCAGGTCGACTCT; GFP-R0 GTGTAATCCCAGCAGCTG. Information about other primers is available from the authors upon request.

### *In situ *hybridization

A partial sequence for the zebrafish poly(ADP-ribose) glycohydrolase cDNA was amplified using primers neuroC1-F1 and neuroC1-R1 and cloned using a Topo TA cloning kit (Invitrogen) to make pDB376. To make antisense RNA probe, pDB376 was digested with *Spe*I and transcribed with T7 RNA polymerase (Promega) and DIG labeling kit (Roche). GFP probe was made by amplifying GFP with 46 base pairs of EF1α promoter from pT2/S1EF1α-GM2 using primers EF1a-F2 and GFP-R0, and cloning it into Topo TA cloning kit resulting in pSS100. pSS100 was linearized with *Spe*I and transcribed with T7 RNA polymerase using DIG labeling kit. To make *mkp3 *antisense probe, *mkp3 *cDNA was amplified from maternal cDNA library with primers Mkp3-F1 and Mkp3-R1 and cloned into Topo TA cloning kit to produce pDB528. The plasmid was linearized with *Spe*I and transcribed with T7 polymerase using DIG labeling kit.

### Screening for germline transmission of *Sleeping Beauty* transposons

Embryos injected with SB10 transposase mRNA and transposon DNA mix were raised as described [32,64]. In pilot screens, adult fish were outcrossed to brass for ease of husbandry. All collected embryos were screened for GFP expression at 1 day post fertilization (dpf) and 3 dpf. We set an arbitrary 200 embryo cutoff for screening, meaning that if less that 200 embryos were obtained from a founder, an additional cross was set up and to obtain additional embryos for screening. Analysis of transgenesis data from pilot screens indicated that 10% of transgenic lines would have been missed if cutoff was set at 100 embryos, and this less stringent coverage protocol was used in scale up screen. Also, we decided to limit screening to 1 dpf since none of the transgenics would have been missed in the pilot screens without the 3 dpf screening.

### Transposon excision in the germline

Homozygous ET1 embryos were injected with SB10 transposase mRNA, raised and screened for loss of hatching gland specific GFP expression, or for a change in the GFP expression pattern. Twenty six fish were screened (R0, for Remobilization), and 2 gave GFP negative embryos, with an additional 2 giving ubiquitously GFP positive embryos, suggesting that germline remobilization events may have occurred in as many as 15% of transposase injected embryos. Ubiquitous GFP positive embryos (one in each of the two R0) did not survive. Of the two R0's that gave GFP negative embryos, one gave mosaic hatching gland expression in the next generation. PCR with transposon specific and flanking primers did not show any changes in the locus. The second R0 produced 19 embryos that were GFP negative from the total of 671 embryos obtainted. An R1 adult was outcrossed, embryo DNA was prepared, and PCR with primers HG1-1 and HG1-2 was conducted. The resulting PCR fragment was cloned using PCR 4 Topo cloning kit (Invitrogen). Plasmids were sequenced using M13 Forward primer, and one clone with a transposon footprint was identified. To confirm that it was not due to PCR contamination, a second clutch of embryos was obtained, the procedure was repeated, and the same footprint was obtained (data not shown).

## Authors' Contributions

The experiments described in this paper were planned, conducted and analyzed as a joint effort between the authors. In particular, DB, AD, SH and ZW contributed to fish screening, line establishment and to scientific descriptions of these lines, DB and SS to molecular analysis, AD and DB to GFP expression and *in situ *analysis. DB designed and built the transposons used in this study and was responsible for drafting the manuscript for publication. SE conceived and supervised the study and edited the manuscript. All authors read and approved the final manuscript.
